# Advancing sarcopenia assessment with wearable and app-based technology: a scoping review

**DOI:** 10.1016/j.jnha.2026.100824

**Published:** 2026-03-11

**Authors:** Ayush Mehra, Justo Perez, Jessica L. Krok-Schoen, Roberto M. Benzo, Colleen K. Spees, Shang-Jui Wang, Steven K. Clinton, Stefan A. Czerwinski, Brett S. Nickerson

**Affiliations:** a College of Medicine, The Ohio State University, Columbus, OH, United States of America; b School of Health and Rehabilitation Sciences, The Ohio State University, Columbus, OH, United States of America; c Ohio State University Comprehensive Cancer Center, Columbus, OH, United States of America; d Department of Internal Medicine, Division of Cancer Prevention and Control, The Ohio State University, Columbus, OH, United States of America; e Department of Radiation Oncology, The Ohio State University, Columbus, OH, United States of America; f Department of Internal Medicine, Division of Medical Oncology, The Ohio State University, Columbus, OH, United States of America

**Keywords:** BIA, Body composition, Skeletal muscle mass, Wearables, Technology

## Abstract

Sarcopenia, a condition characterized by loss of skeletal muscle mass (SMM) and strength, contributes to frailty, disability, and increased healthcare burden, yet remains underdiagnosed due to limitations of conventional assessment methods. Wearable and mobile technologies have emerged as potential tools for SMM evaluation, offering the advantages of portability, low participant burden, and the ability to monitor dynamic changes over time. This scoping review aims to evaluate the current scientific and clinical evidence on wearable and mobile technologies for SMM assessment and to examine their potential application for sarcopenia evaluation in home and non-research clinical settings. Literature searches were conducted across PubMed, Scopus, and Google Scholar in accordance with PRISMA-ScR guidelines. Inclusion criteria comprised original studies reporting SMM as a primary outcome using wearable devices or smartphone-based applications. Review articles and studies without original SMM data were excluded. A total of six studies were included, consisting of three wearable-based investigations and three smartphone application-based studies. Included studies primarily examined apparently healthy or physically active adults, with one study including individuals with type 1 diabetes and overweight or obesity. Sample sizes ranged from 53 to 568 participants, comprising a total of 1,235 individuals across all studies. Overall, studies demonstrated that consumer-grade wearable and smartphone technologies had constant error values (i.e., mean differences) ranging from −0.01 to 4.3 kg, correlation coefficients >0.80, and 95% limits of agreement <±4.0 kg. Criterion measures for comparisons included dual energy X-ray absorptiometry and clinical-grade bioimpedance analysis devices. Altogether, wearable and mobile technologies show emerging potential as accessible tools for SMM assessment and sarcopenia-related monitoring. However, limited study volume, restricted clinical validation, and methodological heterogeneity currently constrain clinical adoption of digital SMM tools, which should be considered adjunctive monitoring instruments rather than diagnostic replacements until rigorously validated across diverse populations.

## 1 Introduction

Sarcopenia is defined as the age- or disease-related loss of skeletal muscle mass (SMM) accompanied by declines in muscular strength and physical performance [1]. Moreover, sarcopenia is increasingly recognized as a central driver of adverse clinical outcomes, including frailty, disability, loss of independence, and premature mortality [2,3]. Importantly, these functional and clinical consequences underscore that sarcopenia is not merely a physiological change of aging, but a progressive muscle disease with considerable public health implications. Although commonly associated with aging, sarcopenia disproportionately affects individuals with diverse chronic health conditions, including type 2 diabetes, kidney disease, neurological disorders, and cancer [4]. Sarcopenia currently affects an estimated 10% of the global population, with older male populations aged >80 years showing prevalence rates of around 50% [5]. The high prevalence rates emphasize the urgency of improving early identification and monitoring strategies. In addition, as global life expectancy increases and the prevalence of chronic disease rises, the burden of sarcopenia is projected to grow substantially. Moreover, sarcopenia contributes to approximately $18.5 billion in direct healthcare costs annually [6,7]. Despite its clinical and economic implication, sarcopenia remains underdiagnosed and undertreated, in part due to limitations in current assessment modalities and lack of awareness among both patients and healthcare providers [8]. Altogether, these factors highlight a critical gap between the recognized clinical importance of sarcopenia and the practical ability to detect and monitor it effectively.

Early definitions of sarcopenia were established using dual-energy X-ray absorptiometry (DXA) and defined the condition as an appendicular lean mass (ALM) more than two standard deviations below the sex-specific mean of a young reference population [9]. However, contemporary consensus frameworks define sarcopenia as a muscle disease characterized by impairments in muscle strength, muscle quantity, and physical performance [10]. This conceptual progression reflects the growing recognition that muscle strength and performance are critical clinical outcomes, yet accurate quantification of muscle quantity remains foundational to diagnosis. Assessing components of sarcopenia, particularly skeletal muscle mass (SMM), remains challenging as gold-standard imaging modalities such as magnetic resonance imaging (MRI) and computed tomography (CT) are costly and require specialized infrastructure [11]. DXA, while commonly used and recommended in clinical and research settings, also requires dedicated equipment and trained personnel, which may limit accessibility outside structured environments [12]. Although these modalities can be repeated over time, their cost, accessibility, and logistical demands limit their feasibility for frequent longitudinal monitoring in routine clinical care. These limitations highlight the need for tools that are easy to use in routine practice, but that can also monitor SMM more frequently in real-world settings.

Recently, wearable and mobile technologies have emerged as body composition tools with the potential of bridging the gap between clinical accuracy and real-world feasibility [13]. The growing availability of consumer-grade smartwatches, bioelectrical impedance devices, and smartphone-based imaging platforms has created new opportunities for more accessible muscle health assessments. For example, these technologies facilitate more frequent, real-time assessment of body composition in everyday environments, including the home and other non-research clinical settings, extending monitoring beyond specialized research or hospital-based infrastructure [14]. By reducing cost, logistical barriers, and participant burden, consumer-grade devices may enable earlier detection of reductions in SMM and more responsive intervention strategies. To date, most validation studies on smartwatch bioelectrical impedance analysis (BIA) and smartphone-based imaging have focused on fat-free mass (FFM), fat mass (FM), and body fat percentage (BF%) leaving the validity of these technologies for estimating SMM largely unestablished [15]. This discrepancy is notable given that SMM remains a key component of all major sarcopenia definitions. While consensus definition of sarcopenia has changed, SMM has consistently been central to its characterization, highlighting the importance of improving how it is assessed. Therefore, the purpose of this scoping review is to (1) summarize the validity of wearable and mobile technologies for SMM measurements; and (2) discuss their potential to evolve as tools to assess and support sarcopenia diagnosis in home and non-research clinical settings.

## 2 Methods

### 2.1 Design

This scoping review was conducted in accordance with the PRISMA-ScR guidelines, which provide a transparent and systematic framework for mapping the breadth of existing evidence and synthesizing key concepts and theoretical perspectives relevant to a given topic [16]. A scoping review methodology was selected due to the broad nature of the research question. Consistent with this approach, the review was not designed to formally appraise the methodological quality of included studies; rather, it aimed to comprehensively capture and characterize the breadth of literature pertaining to the topic of interest.

### 2.2 Eligibility criteria

This scoping review considered experimental, observational, and descriptive study designs in accordance with PRISMA-ScR recommendations [16]. Eligible experimental studies included randomized controlled trials, non-randomized controlled trials, before-and-after studies, and interrupted time-series studies. Analytical observational studies, including prospective and retrospective cohort studies, case–control studies, and analytical cross-sectional studies, were also eligible. Descriptive observational designs, such as case series, individual case reports, and descriptive cross-sectional studies, were additionally considered.

The population of interest included adults aged 18 years and older. No restrictions were placed on clinical status, care setting, or underlying health condition; thus, studies of community-dwelling individuals, hospitalized patients, and other adult populations were eligible. Studies exclusively involving individuals under 18 years of age were excluded. Studies were included if they evaluated or conceptualized wearable devices, mobile devices, or smartphone-based approaches to body composition assessment with explicit measurement of SMM. Studies were excluded if SMM measurement is not a primary outcome. Review articles and other publications without original SMM data were also excluded. Eligible studies must have been published between January 1, 1990, and December 17, 2025, as wearable technologies were not meaningfully developed prior to this period.

### 2.3 Information sources and search

A comprehensive literature search was conducted on December 17, 2025, across PubMed, Scopus, and Google Scholar. The search was performed by the first author (A.M.) and peer reviewed by members of the research team. These databases were selected to ensure broad coverage of biomedical and interdisciplinary literature.

Searches were limited to the title and abstract fields and used a standardized combination of keywords and Boolean operators. Terms related to body composition and bioimpedance (“bioelectrical impedance analysis,” “bioelectrical impedance,” “bioimpedance,” “body composition”) were combined with terms describing digital health technologies (“telemedicine,” “remote health monitoring,” “wearable technology,” “wearable device,” “smartwatch,” “wearable sensor,” “mobile health,” “smartwatch BIA,” “application-based,” “personalized healthcare”) and further restricted to studies explicitly referencing SMM (“skeletal muscle mass”). The complete Boolean search strategy applied across databases is depicted in Table 1.

**Table 1:** Search string conducted on Pubmed, Scopus, and Google Scholar.

Concept	Search terms (Title/Abstract)
Body composition and bioimpedance	"bioelectrical impedance analysis" OR "bioelectrical impedance" OR bioimpedance OR "body composition"
Digital health technologies	“telemedicine” OR "remote health monitoring" OR "wearable technology" OR "wearable device*" OR “smartwatch” OR "wearable sensor" OR "mobile health" OR “wearable health” OR "application-based" OR "personalized healthcare" OR “smartwatch BIA”
Outcome	"skeletal muscle mass"

### 2.4 Selection of sources

Search results were imported into Covidence, a web-based systematic review management platform that facilitates independent screening and study selection by multiple reviewers [17]. Two reviewers (A.M. and J.P.) independently screened all titles, abstracts, and full-text articles for eligibility. Two discrepancies were resolved through discussion between the two reviewers. When consensus could not be reached, a third reviewer (B.S.N.) was consulted to adjudicate inclusion.

### 2.5 Data items

Prior to the coding process, the research team developed, by consensus, a set of key domains of interest based on the objectives of the scoping review and the research question. These domains included (1) consumer wearables and (2) applications. To be included, technologies were required to have the capability to estimate SMM. Eligible items were subsequently reviewed and analyzed by the research team to evaluate their potential to evolve as tools for sarcopenia assessment.

### 2.6 Data charting process

Data were extracted using a standardized charting form that captured key information, including sample size, participant age, population, ethnicity, prediction methods, criterion methods, and SMM findings. The form was iteratively refined using a subset of included studies to ensure completeness and consistency. Two reviewers (A.M. and J.P.) independently charted data from all eligible articles, with one discrepancy resolved through discussion and consensus. When agreement could not be reached, a third reviewer (B.S.N.) adjudicated.

### 2.7 Synthesis of results

Included articles were categorized into one of two predefined domains based on their primary focus: (1) consumer wearables or (2) applications. Each article was assigned to the domain that best represented its main technology or approach for estimating SMM. This categorization allowed the research team to systematically analyze and compare the potential of different technologies for sarcopenia assessment.

## 3 Results

**Fig. 1:**
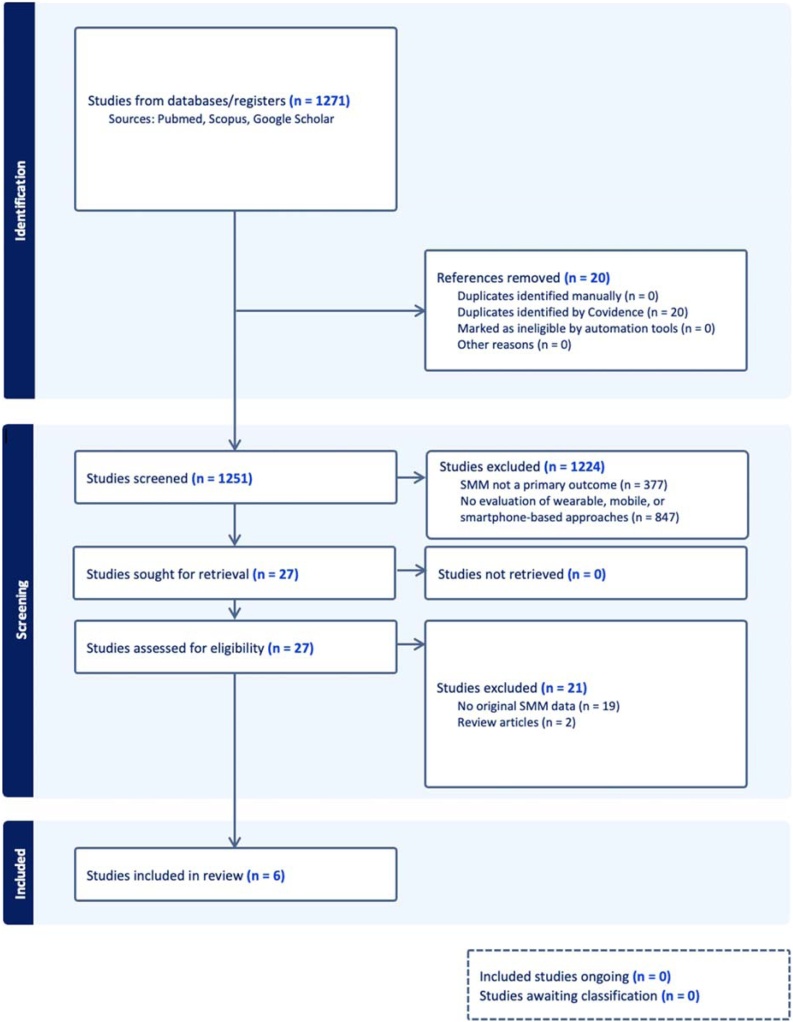
Flowchart for inclusion process.

A flow diagram of the included studies is represented in Fig. 1. The searches extracted 1271 peer-reviewed articles from all electronic databases. Articles that did not meet the inclusion criteria at the title, abstract, or full-text stage were excluded from subsequent steps (n = 1224). Also, articles that did not list SMM as a primary outcome (n = 19) or without original SMM data (n = 2) were excluded. In total, 6 articles were included in the final analysis, which consisted of articles examining the accuracy of SMM estimated from consumer wearables (n = 3) and smartphone applications (n = 3). The articles included in this review were published from 2015 to 2025 and had been conducted in different countries: USA (3) [[18], [19], [20]], South Korea (1) [21], Italy (1) [22], and Turkey (1) [23].

Characteristics and agreement outcomes of digital health devices for estimating SMM for included studies is depicted in Table 2. Across included studies, validation populations primarily consisted of apparently healthy (n = 1018) or physically active (n = 108) adults, with one study specifically examining overweight or obese individuals with type 1 diabetes (n = 109). Sample sizes ranged from 53 to 568 participants, and participant ages spanned young to middle-aged adulthood. Three studies included racially diverse cohorts, with two studies including Asian, non-Hispanic Black, Hispanic, native Hawaiian or Pacific Islander, and non-Hispanic White [19,20], and one study including Asian, Caucasian, and non-Hispanic Black [21]. However, reporting of demographic characteristics was inconsistent as three studies did not provide ethnicity distributions [18,22,23].

**Table 2:** Characteristics and agreement outcomes of digital health devices for estimating skeletal muscle mass in adults.

Device Type	Author	Sample Size	Age (yrs.)	Population	Race/Ethnicity	Digital Health Device	Criterion	Results
Wearables	Manci et al.	Males (n = 199) Females (n = 123)	25 ± 11	Apparently Healthy	Not Provided	InBody Band	BIA (InBody 720)	CE = 0.51 kg *r* > 0.90 95% LOAs <±4.0 kg
	Bennett et al.	Males (n = 34) Females (n = 41)	40 ± 16	Apparently Healthy	Asian NH Black Hispanic NHOPI NH White	Samsung Galaxy Watch 4 Samsung Galaxy Watch 4 Classic	DXA (Hologic Discovery A)	CE = 2.2 kg *r* = 0.98 95% LOAs <±3.5 kg
	Carrier et al.	Males (n = 52) Females (n = 56)	39 ± 14	Physically Active	Not Provided	Samsung Galaxy Watch 5	DXA (GE iDXA)	CE = 4.3 kg *r* = 0.92
Applications	McCarthy et al.	Males (n = 26) Females (n = 27)	39 ± 15	Apparently Healthy	Asian NH Black Hispanic NHOPI NH White	MeThreeSixty	DXA (Hologic Discovery A) (Hologic Horizon A)	*r* > 0.80 RMSE values < 2.0 kg 95% LOAs <±4.0 kg
	D’Abbronzo et al.	Males (n = 56) Females (n = 53)	54 ± 16	Overweight/Obese Type 1 Diabetes	Not Provided	Metadieta	A-wave BIA (Quantum V)	CE = −1.7 kg
	Choi et al.	Males (n = 245) Females (n = 323)	43 ± 17	Apparently Healthy	Asian Caucasian NH Black	Prototype Smartphone	DXA (GE Prodigy)	CE= −0.01 to 0.12 kg *r*-values > 0.90

CE = constant error; SEE = standard error of estimate; RMSE = root mean squared error; LOAs = limits of agreement coefficient DXA = dual energy X-ray absorptiometry; BIA = bioimpedance analysis; NH = non-Hispanic; NHOPI = native Hawaiian or Pacific Islander.

Among wearable-based studies, one study compared a wrist-worn bioimpedance device with a laboratory-grade BIA system [23], while two investigations validated commercial smartwatch-derived SMM estimates against DXA [18,20]. The InBody Band had a constant error (CE) of 0.51 kg, strong correlation coefficients (*r* > 0.90), and 95% limits of agreement (LOAs) < ±4.0 kg when compared against the criterion BIA [23]. Smartwatch-based approaches using the Samsung Galaxy Watch 4 and Galaxy Watch 5 also exhibited strong correlations with DXA-derived SMM (*r* > 0.90) and fairly small CE (2.2 kg) and 95% LOAs (<±3.5 kg) values [20]. The Galaxy Watch 5 demonstrated a similar correlation coefficient as previous studies (*r* = 0.92), but had the largest CE (4.3 kg) when compared to DXA [18].

When examining smartphone-based applications, MeThreeSixty provided moderate to strong correlation coefficients (*r* > 0.80) and low 95% LOAs <±4.0 kg, when compared to DXA [19]. In contrast to most studies reporting similar or slightly overestimated values, the Metadieta application was found to underestimate SMM the most (CE = −1.7 kg) when compared against BIA [22]. Finally, a prototype smartphone-based system demonstrated minimal systematic bias (CE values ranging from −0.01 to 0.12 kg) and strong correlation coefficients (*r* > 0.90) when compared to DXA [21].

## 4 Discussion

The purpose of this scoping review is to (1). summarize the validity of wearable and mobile technologies for SMM measurements; and (2). discuss their potential to evolve as tools to assess and support sarcopenia diagnosis in home and non-research clinical settings. The information analyzed came from 5 cross sectional studies and 1 crossover study, which included 3 wearable and 3 smartphone-based applications. DXA was used as the criterion in 4 studies [[18], [19], [20], [21]] whereas 2 studies utilized a clinical-based BIA device for the reference method [22,23]. Overall, CE values were <2.5 kg across all studies, other than Carrier et al. [18] which observed a systematic overestimation of SMM for the Samsung Galaxy Watch 5 (CE = 4.3 kg). These findings suggest the wearable and smartphone-based applications may be useful for measuring SMM at the group level. Additionally, the Samsung Galaxy Watch 5, when utilized in physical active adults may need correction to account for systematic bias (e.g., decrease SMM approximately 4 kg). However, further research needs to determine whether the bias (i.e., overestimation) occurs in other populations. The correlation coefficients were >0.80 for wearables and apps across all studies. Furthermore, the 95% LOAs were <±4.0 kg for all studies. Collectively, these findings indicate the wearable and smartphone-based applications provide similar group and individual error as field methods (e.g., skinfold thickness, hand-to-foot BIA).

### 4.1 Wearables

The lowest CE values, observed via the InBody Band (CE = 0.51 kg), are likely attributed to the comparison against the InBody 720. The Samsung Galaxy Watch 4 had higher CE values than the Samsung Galaxy Watch 5 (CE = 2.2 and 4.3 kg, respectively), both of which were higher than the InBody Band. Reasons for discrepancies in CE values could be multifactorial. First, DXA was used as a criterion for determining the agreement with Samsung Galaxy Watch 4 and 5. For example, clinical-based InBody devices (e.g., InBody 230, Inbody 720, Inbody 770) have previously been found to yield similar CE values when compared to DXA [24]. Secondly, the Hologic Discovery A was used as a criterion for determining the agreement with the Samsung Galaxy Watch 4; whereas the GE Lunar iDXA was the reference standard for the Samsung Galaxy Watch 5 evaluation [18,20]. Differences in body composition metrics when comparing GE against Hologic DXA models have previously been noted [25]. For example, GE has been shown to yield higher ALM than Hologic (CE = 1.8 kg) devices [26]. Finally, the study sample for the Samsung Galaxy Watch 5 was noted as physically active, defined as participating in moderate to vigorous physical activity at least three days per week [18] while the study sample for the InBody Band and Samsung Galaxy Watch 4 were both considered apparently healthy [20]. The mean age for the apparently health and physical activity study samples were similar (40 and 39 yrs, respectively), but the mean SMM was higher in the former (29.4 and 23.1 kg, respectively). It is unclear whether moderate to vigorous activity was subjectively or objectively determined (e.g., self-reported vs. accelerometry) [18]. Accordingly, comparisons in apparently healthy and physically active populations are needed of further investigation. Additionally, the validity of wearables for estimating SMM in clinical, aging, and diseased populations has yet to be established. Thereby highlighting the need to expand research in this area before widespread adoption of wearable technology.

### 4.2 Apps

The current scoping review demonstrates that smartphone-based applications yielded strong SMM correlation coefficients and low 95% LOAs, similar to results observed via wearables. However, the range in CE values were smaller for apps (CE = −1.7 to 0.12 kg) than wearables (CE = 0.51–4.3 kg). For example, contrary to wearables, the largest CE values for apps were observed when using BIA (Quantum V) as a reference method [22]. It is worth noting that the study population for the app Metadieta had a higher mean age (54 years) and were determined to be overweight/obese with Type 1 diabetes. Moreover, the study sample for the Metadieta app was the only clinical population evaluated in the current scoping review. Accordingly, it is unclear whether CE values are similar in other diseased populations (e.g., sarcopenia, cancer). In contrast to the limited SMM-focused literature, a majority of app-based research to-date has examined anthropometric measures derived from two- or three-dimensional body shape smartphone imaging, which have been subsequently used to estimate outcomes such as BF%, FM, and total body water (TBW). BF% derived from 2D images were compared to DXA and found strong correlations (r = 0.86) among apparently healthy adults (n = 226) [27] and in a separate study demonstrated high overall concordance with DXA among men (concordance correlation coefficient (CCC) = 0.94, n = 52) and women (CCC = 0.93, n = 84) [28]. Similarly, FM derived from 2D images were compared to DXA and found strong correlations (*p* < 0.001) with 95% LOAs for males ranging from −4.9 to 3.9 kg for males (n = 74) and −4.3–4.9 kg for females (n = 84) [29]. Beyond FM outcomes, TBW derived from 3D smartphone images were compared to bioimpedance spectroscopy and found strong correlation (*r* = 0.95, n = 338), however, moderate-to-large 95% LOAs were observed (±6.41 L) [30]. Taken together, these findings indicate that smartphone-based imaging applications demonstrate reasonable performance for fat-related metrics (BF% and FM) based on CE, *r* values, and 95% LOAs, but provide limited evidence for SMM estimation. As such, app-based technologies may not be ready for widespread clinical implementation, and robust validation across diverse healthy and disease-specific populations is required before integration.

### 4.3 Translation to older adults

Many of the studies in this scoping review included relatively young and apparently healthy adults, which may limit the translation of these findings to older populations, which are at greatest risk for sarcopenia. For example, aging is associated with physiological and clinical factors that can influence body composition estimation such as altered hydration status, low-grade inflammation, multimorbidity, and functional decline [31,32]. Accordingly, these issues could impact the measurement assumptions that underly these technologies such as BIA and image-based body composition estimation; thereby, altering device accuracy in older adults. Additionally, age-related changes in fat distribution, muscle tissue, and fluid balance may further complicate estimating SMM with these devices in aging populations [[32], [33], [34]]. Consequently, validation studies designed for older adults and other clinical populations are risk for sarcopenia are necessary to determine whether the findings observed in younger cohorts in the current scoping review can be generalized to individuals most affected by sarcopenia.

### 4.4 Future directions

SMM remains a central component of contemporary sarcopenia frameworks, though diagnosis requires integration of muscle strength and function as well as muscle mass [12]. To date, no studies have used consumer wearable or application-based technologies to assess sarcopenia. Consequently, the diagnostic accuracy of wearable and application-based technologies to diagnose sarcopenia, in place of imaging and bioimpedance-based technology, remains elusive. However, findings from this scoping review suggest that wearable and application-based technologies demonstrate preliminary promise for estimating SMM. Accordingly, future research should integrate SMM estimates derived from wearable and application-based technologies into sarcopenia framework. Digital SMM estimates derived from wearables and applications may function as early indicator or longitudinal monitoring tools within sarcopenia pathways, triggering targeted clinical assessments (e.g., handgrip strength, gait speed) in response to concerning changes. Digital health tools may also offer greater value for population-scale surveillance, remote follow-up, and outcome-oriented trials that track longitudinal trajectories, rather than as direct replacements for DXA, MRI, or CT measures of SMM. Populations undergoing periods of rapid body composition change such as cancer treatment or glucagon-like peptide-1 receptor agonist (GLP-1RA) associated weight loss may particularly benefit from longitudinal digital SMM estimates [35,36]. GLP-1RA therapies often induce rapid weight loss that includes disproportionately high muscle loss, with studies suggesting that 20–50% of the weight lost is lean body mass [37,38]. Continuous wearable and app-based tracking of SMM could flag these early declines, triggering timely interventions to limit muscle loss seen during rapid weight reduction. In addition, combining SMM estimates with other device-measured activity (e.g., step count, sleep metrics) could further inform risk stratification for functional decline, provided these approaches are carefully validated against measures of SMM, strength, and performance. Finally, future work should emphasize rigorous validation of wearable and app-based SMM estimates within sarcopenia frameworks before broader clinical adoption.

### 4.5 Limitations

This review followed PRISMA-ScR guidance and employed dual independent screening and data charting to map the literature comprehensively. However, by design, a scoping review does not formally appraise study quality; therefore, our synthesis emphasizes scope and patterns rather than graded evidence statements. Additionally, several considerations should be acknowledged when interpreting the findings of this scoping review. First, the heterogeneous nature of the included literature limits direct comparison across studies, as substantial variability exists in device types, proprietary algorithms, validation protocols, reference standards, and analytic approaches. Second, the rapidly evolving landscape of consumer digital health technologies must be considered, as device hardware, firmware, and prediction algorithms are frequently updated; consequently, the performance characteristics summarized in this review reflect current implementations rather than permanent or fixed measurement capabilities. Third, population representation remains limited, with most validation studies conducted in younger, healthier adults [[18], [19], [20], [21],^23^] and relatively few investigations in older individuals, hospitalized patients, or populations with chronic disease [22]. Device performance may vary across age, health status, body composition phenotype, and racial or ethnic background, underscoring the importance of inclusive, population-diverse validation efforts [20]. Fourth, most studies were conducted in younger, healthy adults, limiting generalizability to older populations. Age-related factors such as hydration changes, inflammation, multimorbidity and functional decline may influence the accuracy these devices. Finally, this review focused specifically on SMM rather than the full multidimensional construct of sarcopenia, which also includes muscle strength and physical performance. Together, these factors highlight the need for standardized acquisition protocols, transparent algorithm reporting, and rigorous benchmarking against gold-standard imaging methods to support reliable clinical translation of wearable and smartphone-based muscle assessment technologies.

## 5 Conclusion

This scoping review examined the available evidence of wearable and application technologies for assessing SMM against various criterion methods (i.e., DXA and clinical-based BIA). Overall, 6 studies demonstrated that consumer-grade wearable (n = 3) and smartphone technologies (n = 3) tend to produce low group and individual error. Nonetheless, most research to-date has elevated healthy adults with limited focus on clinical populations, other than overweight/obese adults with Type 1 diabetes [22]. Accordingly, there is a need to further explore the accuracy of digital health technologies in racially and ethnically diverse populations, as well as special (e.g., Down syndrome) and diseased populations (e.g., sarcopenia, cancer). It is unknown whether these technologies can be used for tracking changes in SMM longitudinally. Consequently, research needs to explore if these devices are valid for tracking rapid changes in SMM that occur from various clinical treatments such as cancer or GLP-1RA, where treatment-related muscle loss and sarcopenia risk are high. Current evidence is limited and heterogeneous. Furthermore, device algorithms are often opaque, and validations lack breadth across clinically important populations. Consequently, until these gaps are addressed through rigorous, inclusive, and transparent validation efforts, digital SMM tools are best framed as adjunctive screening and longitudinal monitoring instruments rather than replacements for imaging-based diagnostic standards for sarcopenia. Nonetheless, with coordinated methodological improvements, these technologies have the potential to play a major role in the future of sarcopenia research and clinical care.

## CRediT authorship contribution statement

A.M., J.P., and B.S.N. conceived the review topic, performed the literature search, and drafted the manuscript. J.L.K.-S., R.M.B., C.K.S., S.-J.W., S.K.C., and S.A.C. provided additional content, critical revisions, and intellectual input. All authors contributed to editing, reviewed the manuscript for important content, and approved the final version.

## Ethical standards

This article is a review of previously published literature and does not include any new studies with human participants or animals conducted by the authors. Therefore, ethical approval and informed consent were not required. Clinical trial number: not applicable. Consent to Participate declaration: not applicable. Consent to Publish declaration: not applicable.

## Declaration of Generative AI and AI-assisted technologies in the writing process

During the preparation of this manuscript, the authors used ChatGPT (OpenAI) to assist with framework, with the aim of improving readability and clarity. No content from the generative AI was used within the manuscript, and the authors take full responsibility for the accuracy and integrity of the work.

## Funding

Research reported in this publication was supported by the National Institute of Child and Human Development of the National Institutes of Health under Award Number R21HD115191. The content is solely the responsibility of the authors and does not necessarily represent the official views of the National Institutes of Health.

## Data availability statement

This review did not generate any new data. All data discussed are derived from previously published studies available through publicly accessible databases such as PubMed, Google Scholar, etc.

## Declaration of competing interest

The authors declare no conflict of interest.
